# Strategic Planning Insights for Future Science-Driven Extravehicular Activity on Mars

**DOI:** 10.1089/ast.2018.1850

**Published:** 2019-03-06

**Authors:** Allyson L. Brady, Shannon E. Kobs Nawotniak, Scott S. Hughes, Samuel J. Payler, Adam H. Stevens, Charles S. Cockell, Richard C. Elphic, Alexander Sehlke, Christopher W. Haberle, Greg F. Slater, Darlene S.S. Lim

**Affiliations:** ^1^ School of Geography and Earth Sciences, McMaster University, Hamilton, Canada.; ^2^ Department of Geosciences, Idaho State University, Pocatello, Idaho.; ^3^ UK Centre for Astrobiology, School of Physics and Astronomy, University of Edinburgh, Edinburgh, United Kingdom.; ^4^ NASA Ames Research Center, Moffett Field, California.; ^5^ School of Earth and Space Exploration, Arizona State University, Tempe, Arizona.; ^6^ Bay Area Environmental Research Institute (BAERI), NASA Research Park, Moffett Field, California.

**Keywords:** Strategic planning, Analog, Science traceability matrix, Extravehicular activity, Basalt, Habitability, Planetary science, Mars, Human exploration

## Abstract

Short-term and long-term science plans were developed as part of the strategic planning process used by the Biologic Analog Science Associated with Lava Terrains (BASALT) science team to conduct two Mars-simulation missions investigating basalt habitability at terrestrial volcanic analog sites in 2016. A multidisciplinary team of scientists generated and codified a range of scientific hypotheses distilled into a Science Traceability Matrix (STM) that defined the set of objectives pursued in a series of extravehicular activity (EVA) campaigns performed across multiple field deployments. This STM was used to guide the pre-deployment selection of sampling stations within the selected Mars analog sites on the Earth based on precursor site information such as multispectral imagery. It also informed selection of hand-held instruments and observational data to collect during EVA to aid sample selection through latency-impacted interaction with an Earth-based Science Support Team. A significant portion of the pre-deployment strategic planning activities were devoted to station selection, ultimately the locations used for sample collection and EVA planning. During development of the EVAs, the BASALT science team identified lessons learned that could be used to inform future missions and analog activities, including the critical need for high-resolution precursor imagery that would enable the selection of stations that could meet the scientific objectives outlined in the STM.

## 1. Introduction

Crewed missions to Mars will demand focus on integrating scientific return and sample collection activities into planetary mission concepts. Although future astronauts are likely to have more scientific training and experience in the field specific to the search for life as compared with those on the Apollo missions (Lofgren *et al.*, 2011), well-informed identification of potential sample sites and rigor in sample collection for multifaceted analyses in the fields of biology, geology, and geochemistry is critical to the success of any coordinated astrobiological investigation. Advanced traverse planning and site selection guided by well-developed scientific objectives and hypotheses for future human missions will be fundamental to enabling scientific productivity (Eppler, 2011).

At the same time, extravehicular activity (EVA) must allow for flexible adaptations to observational data that may impact scientific hypotheses and questions, and a degree of astronaut autonomy in the identification of representative scientific samples. In addition, planning must allow for latency-impacted interaction with Earth-based teams of scientific experts located in a Mission Support Center (MSC) (Eppler *et al.*, 2013). Communication latencies between Mars and Earth will introduce new challenges to scientific collaboration between the MSC-based science backroom and extravehicular/intravehicular (EV/IV) crewmembers. Future human missions to Mars are likely to be long, complex, and include scientific objectives (Drake, 2009a, 2009b), necessitating an understanding of how best to facilitate these interactions to enable science productivity.

Strategic planning, defined as the generation of mid-term and long-term science plans used to direct EVAs conducted over a mission or multiple missions (Eppler *et al.*, 2013), is an essential aspect of crewed science-driven space exploration. For the Mars Exploration Rovers (MER) *Spirit* and *Opportunity*, strategic planning involved remote-sensing data throughout a 2+ year landing site selection process, where multidisciplinary members of the scientific community were solicited for input to identify locations that would not only meet engineering constraints but also provide opportunities for scientifically valuable research (*i.e*., potential lacustrine settings at Gusev Crater) (Golombek *et al.*, 2003, 2005). The use of science objectives as the final discriminator between MER sites contrasted with previous missions such as *Viking* that focused on engineering constraints (Golombek *et al.*, 2003).

During strategic mission planning and site selection, proposed landing sites were prioritized according to scientific potential as well as safety, resulting in the ultimate selection of two sites that met the overall mission objectives of addressing science questions related to past environmental conditions and water activity (Squyres *et al.*, 2003). For the finalist MER landing sites, scientists attended multiple workshops where teams generated site-specific lists of testable hypotheses for the identified sites of interest (Golombek *et al.*, 2003).

Strategic planning must also be considered not only before a mission but potentially during one as well. During the MER mission, the science team used the Science Activity Planner (SAP) to direct the activities of the rovers. The SAP enabled data analysis and construction of planned traverses and science activities that would be executed on the following sol (martian solar day) based on the information gathered and processed by the science team (Norris *et al.*, 2005). This allowed the science team to respond to new scientific data and insight during the deployment. Robotic operations planning requires the strategic planning process to ultimately derive the set of exact commands to be scripted and sent to the assets for execution. However, adding humans changes the paradigm as scientific decision making during an EVA may then occur on both sides of the communications latency. As such, the current strategic planning process must adapt to incorporate the expertise, intuition, and decision making of future human spaceflight missions and potentially allow for scientific-based strategic decisions during an EVA.

The only historical precedent of human spaceflight strategic scientific planning occurred during lunar surface Apollo missions. Scientific interests were collected and categorized through a multiyear series of workshops and meetings into three basic problem areas to address (1) the structure and processes of the lunar interior, (2) the composition and structure of the surface of the Moon and the processes modifying the surface, and (3) the history or evolutionary sequence of events by which the Moon has arrived at its present configuration (Compton, 1989). These categories were ultimately distilled into 15 primary research questions that influenced the specific activities and experiments performed on the Moon (NRC, 1966). This strategic science development effort was supplemented throughout the Apollo missions by the inclusion of “Surface Working Groups” (SWG) that provided recommendations for planning and procedure development as well as relevant real-time guidance during each EVA (Schmitt *et al.*, 2011).

However, the Apollo mission objectives were fairly geological in nature as at the time of the missions, relatively little was known about the lunar surface and astronauts were tasked with creating a baseline level of contextual knowledge (Clark, 2010). Although revolutionary in the inclusion of communication with a backroom of science (geology) experts on the Earth, the Apollo missions were strictly scheduled, leaving little room for exploration and the SWG had little opportunity to alter the EVA in response to any scientific discoveries. Even in the case of the Apollo missions, the need for scientific input, and potential value added by including scientist-astronauts, was recognized and suggested (*e.g*., Sonnet, 1963).

Future planetary exploration, including crewed missions to Mars, is expected to emphasize scientific exploration and observation, as well as acquiring data and samples of high scientific value to a much greater degree than was experienced on Apollo (Hurtado *et al.*, 2013). As the amount of material that may be returned to the Earth for scientific analysis will be limited due to mass restrictions (Moores *et al.*, 2012), sample suites will need to be carefully selected and triaged (assigned a relative value or priority) during the mission to select the most representative samples. We envision that such missions will be influenced by terrestrial field research approaches from a multitude of scientific disciplines such as geophysics, geochemistry, and astrobiology, with the EVA crew acting as an extension of a multidisciplinary science support team (SST) on the Earth (Drake, 2009a, 2009b; Yingst *et al.*, 2013).

As it is anticipated that early findings will influence subsequent activities, the science team will be responsible for tracking mission accomplishments, and re-evaluating EVA objectives as new discoveries are made; this will require inter- and intra-EVA strategic decision making, building on the larger strategic plan established before the mission. Although integration of an Earth-based SST has been explored in past analog programs such as the Pavilion Lake Research Project (PLRP) (Lim *et al.*, 2011) and NASA Extreme Environment Mission Operations (NEEMO) (Chappell *et al.*, 2016), there are still outstanding questions with respect to integration. There remains a knowledge gap in our understanding of (1) how best to integrate disparate multidisciplinary science objectives, (2) adaptations to new data both during and between EVAs, and (3) methods of integrating scientific experts. The strategic planning required to execute human EVAs necessarily must integrate these aspects and address associated challenges, providing an opportunity to gain insight into the factors that play an important role.

Here, we describe the strategic planning process used during the Biologic Analog Science Associated with Lava Terrains (BASALT) program to conduct non-simulated scientific research into basalt habitability under Mars-simulated conditions that address this knowledge gap. The science objectives and activities conducted as part of the BASALT program are considered “non-simulated” in that they contribute fundamental biological and geological knowledge regarding microbial habitability with relevance outside of the analog program. An overview of the BASALT project is presented in the work of Lim *et al.* (2019), whereas the specific concepts of operation (ConOps) tested are presented in the work of Beaton *et al.* (2019a). The geology of the two high-fidelity Mars analog sites explored, Craters of the Moon National Monument and Preserve (COTM), Idaho and Hawai‘i Volcanoes National Park (HVNP), is described in the work of Hughes *et al.* (2019).

The BASALT research program explored both strategic and tactical requirements designed to facilitate science-driven interaction across communication latencies representative of Earth–Mars one-way light time; *tactical* refers to intra-EVA interactions regarding science objectives within a given EVA, whereas *strategic* refers to interactions regarding overall progress of the mission science objectives and planning for future EVA traverses, which may happen pre-mission, pre-EVA, intra-EVA, and/or inter-EVA. We investigated how effective strategic planning in advance of planetary missions could be supported through the collection of critical precursor information and data, including high-resolution imagery and remote-sensing data, and used to enable valuable observational data/sample collection within the operational confines of a Mars EVA.

Creation and use of these data products requires an understanding of the type of information that is most valuable for meeting the scientific objectives of the overall mission or specific EVA. The management and evolution of strategic aspects of the BASALT program, that is the generation of both long-term and short-term scientific goals, are the focus of this article. Here, the use of “long-term goals” refers to the overall science objectives of the program, spread across multiple deployments, whereas short-term refers to specific EVA (*e.g*., daily) objectives. Tactical elements related to the execution of EVAs and activity of the science team during an EVA are discussed in the work of Stevens *et al.* (2019), the layout and functioning of the Earth-based MSC and SST is described in the work of Payler *et al.* (2019), and modes and frequency of communication between the SST and EV/IV are presented in the work of Kobs Nawotniak *et al.* (2019). This article presents and discusses the science organization and prioritization process needed to strategically plan science-driven EVAs and associated timelines that supported full duplex, Mars latency-tolerant operational communications scenarios at BASALT. This includes:

(1) Articulation of mission and EVA science objectives
(2) Technical requirements generation (*e.g*., software and hardware, orbital data)
(3) Common language (*e.g*., geological terms and spatial terminology)
(4) Task articulation (EVA activities and scientific tasks).

## 2. Methods: Science-Driven EVA Planning and Execution During the BASALT Campaign

### 2.1. Articulation of BASALT science objectives and creation of a Science Traceability Matrix

Articulation of the BASALT campaign science objectives began at the proposal stage with the identification of overarching research questions of interest to the BASALT science team as informed by previous analog, habitability, and biosignature research (see Lim *et al.*, 2019 for an overview). Terrestrial basalt environments have been shown to host heterotrophic and chemolithic bacteria that use iron and sulfur as energy sources (Herrera *et al.*, 2009; Kelly *et al.*, 2014). Widespread volcanism on Mars could have led to the creation of habitable environments as basaltic rocks underwent alteration. Increased porosity and secondary minerals could provide new energy sources for organisms, leading to increased microbial colonization (Cousins, 2015). However, analog studies examining microbial populations in volcanic terrains on the Earth and how microbial growth, diversity, and potential generation of biosignatures relate to the geochemical and geological conditions are lacking.

The BASALT research program combined both geological and biological approaches to address the overarching question: How do microbial communities and habitability correlate with physical and geochemical characteristics of chemically altered basalt environments (Lim *et al.*, 2019)? With respect to future Mars mission planning, these combined approaches address a critical need to determine the relationship between mineralogical/geochemical parameters that can be measured from orbit with the potential for high scientific return, specifically, with respect to the potential for detection of enabling efficient and effective selection of sampling sites.

Because the BASALT program was driven by real, non-simulated science questions, a Science Traceability Matrix (STM) was developed by the multidisciplinary science team that was foundational to the subsequent EVA planning and selection of observational data to be acquired by EV crewmembers during the two 2016 deployments (Table 1). An STM provides an overview of what a mission will accomplish and formalizes and communicates science requirements in a manner that moves from broad, high-level questions to more specific hypotheses and tasks (Jones-Wilson and Susca, 2017; Susca *et al.*, 2017). For example, the planned Europa Mission has integrated an STM as part of the strategic mission planning and the foundational development of science objectives and associated tasks (*e.g*., Jones-Wilson and Susca, 2017; Susca *et al.*, 2017). The BASALT science team comprised experts in biology, geochemistry, and geology. Each research group had specific objectives and hypotheses related to habitability of basalts that fit within individual areas of expertise and analytical capabilities. As part of the early strategic planning, the science team worked together to identify common sample types, locations, and quantities that would enable collaborative, complementary analyses and data-driven publications. A series of weekly telecons provided opportunities for extensive discussion and input from all scientists.

**Table 1.** BASALT Science Traceability Matrix Demonstrating High-Level Science Questions Related to Basalt Habitability and the Specific Hypotheses and Tasks to Be Tested and Executed During the 2016 Deployments

*First-order question*	*Specific objectives and questions*	*What do we want to know?*	*Associated hypotheses*	*Proposed 2016 priorities and science tasks*
How do microbial communities and habitability correlate with the physical and geochemical characteristics of chemically altered basalt environments?	Geo1: What are the geochemical, mineralogical, and textural properties associated with basalts affected by liquid water, intrinsic volatiles, and fumarolic gases at complementary Mars analog sites?	(A) Which mineral assemblages (primary and secondary from deposition and/or alteration) occur in relation to each of the water alteration mechanisms (liquid, intrinsic volatiles, fumaroles)? How do the mineral assemblage ratios change across the alteration gradient, and what scale does that occur on? How are those gradients changed by variation in parent rock geochemistry? Do changes in macro-texture of the rock (scoria/pahoehoe/slabby pahoehoe/etc.) produce significant changes in opportunity for water persistence and, therefore, alteration?	(HA1) Subsequent secondary alteration from atmospheric and/or groundwater will differently affect products formed from intrinsic volatile alteration.	(A1) Collect samples from volatile/fumarole (HVNP) and liquid water-dominated alteration areas for mineral analyses.
	Geo2: What geochemical and geological conditions provide appropriate energy sources, major biogenic elements (CHNOPS), liquid water, and micro-habitats for microbial growth?			
	Bio1: What is the relationship between the physical characteristics and geochemistry of Mars analog basalts and the biomass they can support?		(HA2) Gradients for liquid water and intrinsic volatile alteration will be relatively gradual (over 10's m), with small pockets of enhanced alteration due to water persistence; fumarolic gradients will be sharp (over 1's m).	(A2) Collect samples along gradients from peak alteration through zero alteration for each alteration type identified in the field. Note: may require sampling inside of pahoehoe blisters and very dense sample spacing on a few key sites.
	Bio2: What are the upper bounds on the biomass that could have been supported on Mars?			
	Bio3: How does this upper bound inform future requirements to detect extinct life on Mars?			
	Bio4: What organic biosignatures are detectable in basalts?			
		(B) To what extent do the different alteration environments (liquid water, intrinsic volatiles, fumarolic gases) change bulk geochemistry of the rock? Does the relatively dry alteration at COTM result more in large bulk geochemical changes or in *in situ* reorganization of molecules?	(HB1) Fumarolic alteration will produce the largest changes in rock bulk chemical composition, followed by intrinsic volatile alteration. Liquid water alteration will have the least impact on bulk rock chemistry.	(B1) Same as A1; can use part of sample for geochemistry analyses.
			(HB2) Addition of foreign, wind-blown, or volatile-borne components will produce the greatest chemical and mineralogical variation.	(B2) Collect older rocks.
		(C) How do the different alteration types, and their degrees, influence the microtexture of the rock? Does style of alteration significantly change rock permeability and/or porosity in a way that will enhance microbial community access? Across what kind of gradient? Does degree of alteration within a style significantly change rock permeability and/or porosity? Across what kind of gradient?	(HC1) Temperature and duration of alteration will positively correlate with creation of pathways in the rock for bio-access.	(C1) Same as A1.
			(HC2) Increased alteration will result in increased permeability/porosity.	(C2) Same as A2.
		(D) Which of the combinations just described produces the highest volumes of iron and sulfur oxides that can be accessed by endolithic microorganisms?	(HD1) Hot alteration (volatiles/fumaroles) will result in larger volumes of iron and sulfur oxides than cold alteration.	(D1) Same as A1.
		(E) How do the microbial community structures correlate to the degree of alteration with respect to metabolisms/functional groups? What metabolisms are present in the rocks and do the types of metabolisms correlate to the types and chemical nature of the alterations?	(HE and HF1) More alteration results in greater mineral diversity, which itself allows for greater microbial diversity.	(E–G, I) Collect basalts representing end-members of alteration for organic biomarker analysis: At least six gradients reflect some spatial variability (*i.e*., Big Craters and Highway Flow).
		(F) How does the (species) diversity of microbes in the rocks correlate to the geochemistry and degree of alteration? Does diversity change according to the geochemistry and alteration? Are more altered rocks more or less diverse? Do lipid profiles vary according to the geochemistry and alteration?	(HE2 and HF2) Increased mineral diversity corresponds to increased microbial phospholipid fatty acid diversity.	
		(G) Does total biomass correlate to the diversity of the community and degree of alteration? Are there more or less microbes per unit volume in different rocks with different types of alterations? Does biomass (as measured by lipid content) vary with degree of alteration? Do molecular and organic biomarker analyses generate the same estimates of viable cells?	(HG1) More alteration generates more available elements that allow for greater biomass.	
			(HG2) Detectable levels of organic biomarkers are generated by microbial colonization of the basalts.	
			(HG3) A greater degree of aqueous alteration results in greater microbial biomass as measured by lipid content.	
			(HG4) Molecular and organic biomarker estimates of viable cell counts will differ.	
		(H) Does the presence of iron and sulfur compounds in the rocks influence the presence of chemolithotrophs? (long term). How does the chemical nature of the alteration and the presence of compounds such as iron and sulfur change the ratios of different metabolic groups such as heterotrophs and chemolithotrophs?	(HH1) Rocks containing higher amounts of iron and sulfur compounds harbor a greater diversity of iron and sulfur-using chemolithotrophs.	
		(I) Are organic biomarkers with the potential for long-term preservation (*e.g*., glycolipids) present in basalts?	(HI1) Organic biomarkers with the potential for long-term preservation are present in basalts.	
			(HI2) A greater proportion of biomarkers with higher preservation potential relative to those reflecting viable cells will be observed in rocks that have undergone minimal alteration.	

COTM, Craters of the Moon National Monument and Preserve; HVNP, Hawai‘i Volcanoes National Park.

Before the BASALT-1 deployment to COTM (Idaho), the science team identified the specific scientific questions and hypotheses that fit within the overall BASALT project objectives of investigating habitability in terrestrial basalts (Table 1) (Lim *et al.*, 2019). Broad mission goals were refined into specific questions and objectives and, in turn, distilled into actionable tasks conducted during the individual deployments within the volcanic terrains of interest. The first stage of planning identified high-level questions related to basalt habitability and how geological/geochemical properties may influence microbial colonization (Table 1, columns 1 and 2). More specific questions that arose from each discipline were used as discussion points (column 3) and further refined into testable hypotheses over time (column 4) that could be addressed by collecting samples from both analog environments. These hypotheses were then distilled into specific objectives with respect to data and sample collection during the deployments (column 5).

Due to the multidisciplinary nature of the science team, some research questions overlapped whereas others were specific to the research group. The STM evolved over time as telecons were used to identify common questions and select rock alteration types that could enable the testing of hypotheses generated. In some cases, hypotheses could be tested at both COTM and HVNP (*e.g*., HA1) whereas others could only be tested at one of the field sites (*e.g*., HB1 as fumaroles were only present at HVNP). It was during this time that the science team also determined what additional information would be necessary to collect during the EVA to not only identify the alteration features but also use post-deployment to enable interpretation of analytical research results based on the STM hypotheses. As such, the dynamic STM fundamentally influenced the following aspects of the BASALT campaign:

(1) Mission Planning: selection of region (km-scale), station (10–100 m diameter), and sample location (1 m-scale) interests (Hughes *et al.*, 2019); identifying specific EVA objectives.
(2) Field Instrument selection: identification of hand-held instruments to enable efficient evaluation and grading (“triaging”) of samples in the field (Sehlke *et al.*, 2019).
(3) Laboratory Analyses: documenting the type and rationale for laboratory analyses that ultimately informed the number and manner of sample collection during EVAs.
(4) Science-driven EVA Strategy: the design of scientifically driven EVA phases as informed by the STM, including sequencing, timings, and tasking associated with these phases as well as the definition of roles and selection of personnel (Beaton *et al.*, 2019a).
(5) Science-driven MSC design: physical placement of personnel in the control center, identification of key roles, and the selection of appropriate personnel to fill each role (Payler *et al.*, 2019; Stevens *et al.*, 2019).
(6) Science information needs to support execution of EVA: generation of a list of technical mission requirements and supporting capabilities aimed at meeting research needs that may be used to refine future deployments (Beaton *et al.*, 2019a; Marquez *et al.*, 2019).

As an example, one high-level question outlined in the STM was:

How do microbial communities and habitability correlate with the physical and geochemical characteristics of chemically altered basalt environments?

From this high-level, first-order question, individual research groups developed specific questions based on their area of expertise that would contribute to addressing this overall question of habitability within basalts (*e.g*.,):

Geo1: What are the geochemical, mineralogical, and textural properties associated with basalts affected by liquid water, intrinsic volatiles, and fumarolic gases at complementary Mars analog sites?

Bio1: What is the relationship between the physical characteristics and geochemistry of Mars analog basalts and the biomass they can support?

From these research questions, specific hypotheses to be tested during the 2016 deployments were then generated and sampling activities were outlined that would enable the science teams to address these hypotheses. Two example hypotheses from 2016 are:

(HA2) Gradients for liquid water and intrinsic volatile alteration will be relatively gradual (10 m), with small pockets of enhanced alteration due to water persistence; fumarolic gradients will be sharp (1 m).

(HH1) Rocks containing higher amounts of iron and sulfur compounds harbor a greater diversity of iron and sulfur-using chemolithotrophs.

The necessary contextual information and sampling activities required to address these hypotheses were used by the science team to strategically determine the type of observational data to be gathered during an EVA, as well as to plan the EVA routes and targeted sampling locations with the highest probability of containing representative basalt samples required to meet the outlined scientific objectives. It was also used by the science team to strategically order the EVAs to ensure that collection of high-priority samples was scheduled early in the deployment. The BASALT strategic planning process is further outlined in the following sections.

### 2.2. Technical requirements to enable science productivity

Defining the manner and type of observational information collected that would facilitate scientific decisions was a central component of the BASALT strategic planning process. It also provided an opportunity to learn how to best incorporate new and advanced technologies into more traditional field work practices to enable science activities. Technical requirements were generated by the science team, using the STM as a foundational document that linked science needs to engineering designs (Marquez *et al.*, 2019; Miller *et al.*, 2019) that were employed during the tactical execution of the EVAs (Stevens *et al.*, 2019). For example, the BASALT science team determined, before the initial deployment, that the following capabilities were necessary during the EVAs to enable the science objectives:

(1) Constant commentary (over latency)
(2) Constant video (over latency, high bandwidth conditions)
(3) High-resolution still images (both contextual and close-up of outcrops with scale)
(4) *In situ* geochemical analysis (hand-held instruments).

Technical requirements, including the ability to stream video and voice communications, as well as to deliver data products such as still images, back to the MSC-SST during EVA were identified early on as components that were critical to the success of the BASALT campaign based on the STM. Not only are these data products necessary for tactical responses and evaluation of the proposed sample sites during EVA (Stevens *et al.*, 2019), but they are also a critical component of the context information that the science team will use post-deployment during sample analysis and interpretation. Crew backpacks designed to support the communication of these important data products between EV crew and the MSC-SST are described in further detail in the work of Miller *et al.* (2019). Likewise, the need for a software platform that would enable access by the SST to data products such as images, both before and during the deployments, as well as providing a tool for EVA traverse planning and monitoring was identified.

*Minerva* is an integrated software platform that incorporates tools developed in previous analogs used for both strategic and tactical elements of the BASALT deployment (Marquez *et al.*, 2019). Within *Minerva*, precursor mission data access and EVA traverse planning took place via the eXploration Ground Data System (xGDS) platform (Deans *et al.*, 2017; Marquez *et al.*, 2019). The centralized *Minerva* platform containing the digital precursor information facilitated online collaborative planning among SST members located in various countries and across multiple time zones and was heavily referenced in real time during science team planning telecons. It was also a critical component of scientific data collection (“digital field notebook”) during EVA execution (Marquez *et al.*, 2019; Payler *et al.*, 2019; Stevens *et al.*, 2019).

COTM and HVNP were selected based on existing knowledge of the volcanic processes within these zones that makes them robust analogs for Mars (see Hughes *et al.*, 2019, for more detailed description of the field sites). Within these zones, orbital precursor data (*e.g*., visible wavelength aerial imagery) was identified as a critical need for BASALT strategic mission planning and the selection of stations of scientific interest where the rock samples would be collected. For the BASALT 2016 deployments, station selection began with the collection of remote-sensing data in 2015 with subsequent planning in early 2016 for field activities that occurred in two 10-day periods in 2016 (BASALT-1 Idaho—June 2016 and BASALT-2 Hawai‘i—November 2016). Map layers containing remote-sensing data were imported into the *Minerva* platform and used by the scientists to identify targeted locations that would be used to achieve specific scientific objectives (Marquez *et al.*, 2019) (see Section 3.1). The remote-sensing data used for EVA planning and comparable Mars datasets are listed in Table 2.

**Table 2.** Remote-Sensing Datasets Used to Design BASALT 2016 Field Deployments, Select Regions of Interest, and Determine Applicability of Comparable Datasets for Mars

*Source*	*Resolution (m/pixel)*	*Spectral range (nm)*	*Remarks*
Earth			
Earth Landsat/Copernicus	∼0.15	400–800	Panchromatic, multispectral
Earth Digital Globe^®^ WorldView-3	0.31, 1.24	450–800, 400–1040 (8 bands)	Panchromatic, multispectral
Earth UAV mosaics	0.02–0.05	400–800	Georeferenced orthophotos, DTMs
Earth Aerial LiDAR	0.05–0.30	1047	Digital topography
Mars			
MGS Mars Orbiter Camera Narrow Angle Camera	1.4–12	500–900 (greyscale)	Uses color filter
MGS Mars Orbiter Camera Wide-Angle Camera	230+	400–450 (blue), 574–625 (red)	Two separate cameras with filters
MGS Mars Orbiter Laser Altimeter	∼300 (∼1 m vertical)	1064 near-IR laser	Digital topography
Mars Reconnaissance Orbiter HiRISE	0.3	400–1000 (3 bands)	Multispectral
Mars Odyssey THEMIS	19, 80	Visual (5 bands), infrared (10 bands)	Multispectral, thermal infrared
Mars Express HRSC	2–10	675 (pan), 440 (blue), 530 (green), 750 (red), 970 (near-IR)	Five color filters, spectral values are midpoints

Some imagery obtained from Google Earth^®^ and Bing^®^, both of which use various data sources from Landsat and Earth Digital Globe. Earth Aerial LiDAR data obtained from OpenTopo.

DTM, Digital Terrain Model (topography); IR, infrared; MGS, Mars Global Surveyor; UAV, Unmanned Aerial Vehicle (drone).

Based on the hypotheses generated in the STM regarding the impacts of varying geochemical characteristics on microbial growth and diversity (Table 1), it was determined by the BASALT science team that in-field assessment of rock geochemistry was important to the successful identification of alteration features. More specifically, the *in situ* spectroscopy data were deemed of high priority because they would allow for the identification of alteration properties not readily visible from aerial imagery and/or photography and facilitate assessments of sample alteration state and degree of alteration to identify appropriate end-members (*i.e*., spectral properties that closely matched the alteration features of interest) (Sehlke *et al.*, 2019). These data would also enable the MSC to accept, prioritize, or reject potential candidate sample locations based on the incoming results (Payler *et al.*, 2019; Stevens *et al.*, 2019) during the BASALT deployments. The scientists selected three different instrument types for use during the deployments. One was a forward-looking infrared (FLIR) camera that provided thermal images, critical in identifying active fumaroles within specified temperature ranges and thus only employed in Hawai‘i. The other two instruments were used primarily to triage samples in the field with respect to alteration: a visible-to-near infrared spectrometer that provided identification of minerals (ASD TerraSpec Halo^®^ portable UV/VIS/NIR spectrometer) and an X-ray Fluorescence (XRF) spectrometer that provided elemental composition of the target (Bruker Tracer IV^®^). The application and evaluation of these instruments is presented in the work of Sehlke *et al.* (2019).

### 2.3. Common language

As scientists of varying disciplines and engineers often use distinctive language and unique terminology, it was necessary to implement formal project definitions for terms that would be used to plan and execute BASALT EVAs. This set of definitions included both references to spatial locations, used in articulating EVA tasks, and geological/environmental terms that were used to describe the scientific objectives of each individual EVA and the alteration types being targeted. Project specific spatial terminology is described in Table 3.

**Table 3.** Spatial Terminology Used During BASALT Extravehicular Activity Planning

*Term*	*Spatial scale*	*Definition/example*
Zone	O (100) km	COTM, HVNP
Region	O (1) km	Highway Flow (COTM), Mauna Ulu (HVNP)
Station	10-m diameter (2016 Idaho and 2016 Hawai'i) ca. 100-m diameter (2017 Hawai'i)	An outcrop, deposit, or small cluster of features having overall similar macroscopic characteristics
Sample location	ca. 1-m diameter	Collection location of one set of samples (various replicates) within a station
Sample suite	Variable, within ca. 1-m diameter sample location at each station	Seven rocks collected within ca. 1 m sample locations representing different disciplines
Replicate (each sample)	Precise location of one physical sample	Within the sample location, ca. 1-m diameter

To facilitate complementary analyses across disciplines, rock types were grouped into high-level categories based on alteration type that would enable collection of samples from each category to address multiple hypotheses in the STM. Geological terms used to describe and identify alteration types and features of interest were defined, and example imagery provided, in an Observational Training Document created by the science team to ensure that all members of the MSC and EV/IV crew were familiar with the terminology used in outlining the scientific objectives and priorities. Here, specific geological features of interest that would be used by the MSC-SST to select sample locations were described and instructions were provided for the EV crewmembers with respect to identifying and describing important characteristics that would allow categorization of the rock based on expected alteration or lack thereof. For example, color was used to visually indicate the presence of oxidized iron-bearing minerals whereas texture (*e.g*., porphyritic, aphanitic, glassy) may be used to indicate past or present volatile conditions. Hypotheses within the STM targeted one, or multiple alteration types. For example, hypothesis HA1 in the STM was that “Subsequent secondary alteration from atmospheric and/or groundwater will differently affect products formed from intrinsic volatile alteration.” Atmospheric and/or groundwater alteration and intrinsic volcanic alteration reflect two distinct categories of alteration that may be identified by using visual and/or instrument (spectroscopic) observations. To test this HA1 hypothesis and make this comparison, basalt rocks representative of these two categories were required: rocks that showed a high degree of syn-eruptive alteration (hot, high temperature) and rocks that showed a high degree of atmospheric/groundwater alteration (cold, low temperature). A focus was thus on finding end-member samples for each alteration type (*i.e*., the most/least altered within a given station). The characteristics and criteria of the alteration types of interest are described in greater detail elsewhere (Hughes *et al.*, 2019) but in brief were: (1) unaltered basalt; (2) hot, high temperature alteration (syn-emplacement); (3) cold, low temperature; (4) active fumaroles (temperatures >70°C); and (5) relict fumaroles. A field glossary cuff guide worn by the EV crew is illustrated in the work of Stevens *et al.* (2019).

### 2.4. Specifying granular details from the STM

Although overall mission scientific questions and hypotheses were outlined in the STM, we also developed specific priorities to be pursued on a day-by-day basis during our two field deployments to enable the search for outcrops that met science objectives (Hörz *et al.*, 2013; Hurtado *et al.*, 2013). Within the BASALT framework, this articulation involved identifying baseline sampling requirements, optimal sampling requirements, and, importantly, the observational data products that would provide important contextual information about the station, necessary for future analytical result interpretation, as well as detailed information regarding proposed sampling locations to the MSC-SST during the EVA (see Stevens *et al.*, 2019).

#### 2.4.1. Sampling requirements informed by the STM

Hypotheses generated as part of the STM were distilled into target alteration features that could be identified and sampled during EVAs executed across the BASALT campaign. For example, activities associated with the hypotheses outlined in Section 2.1 would involve identification and collection of basalt rocks that exhibited distinct types of alteration within a station (as per the example of HA2), and basalts with end-member concentrations (high and low) of iron and sulfur (as per HH1), represented by syn-emplacement and unaltered basalts, respectively, to enable comparisons that would test the example hypotheses. A unique challenge that arose due to the multidisciplinary nature of the science team was the variable field practices with respect to sample handling and analytical needs (*e.g*., minimum sample size requirements vary between disciplines, sterility): If the mass of rock sample that can be collected is limited, how best to design EVAs that will allow for multiple questions to be answered across disciplines? It was determined by team consensus that the best course of action was for each EVA to have a primary objective of collecting one defined suite of samples from a given station that would allow for collaborative comparisons of factors and aid in interpretation of individual team results, for example, interpretation of biomass distribution and microbial community profiles are informed by results of the geological description and analysis.

To define the suite of samples that would be collected at a station, the science team considered a wide range of interlaced sample mass and size requirements and analytical restrictions. These acknowledged the fact that some of the different analytical methods have a minimum mass and/or maximum or minimum size requirement; biological analyses require a high level of sterility to prevent contamination, and, for example, each team could only be reasonably expected to curate and analyze a given number of samples over the course of the BASALT project. The suite protocol that defined a full sampling suite at any one location would comprise seven individual rock samples collected with sterilized field equipment and best practices for geological and biological sampling. The seven samples were: three biology samples (DNA extraction), one geology (*e.g*., thin section), two organic geochemistry (biomarkers), and one archive (for curation) that were then distributed to the appropriate members of the science team for post-deployment analyses. All seven samples were to be collected within one 1 × 1 m sampling location (Table 3). This scale was selected to minimize heterogeneity between samples in a suite and, therefore, enable comparative analyses between the different disciplines (Gentry *et al.*, 2017).

Understanding the spatial variability that exists within similar environmental/sampling types (*e.g*., active fumarole) is critical when life- or biosignature detection is the primary scientific objective. If small-scale variability is high and not well constrained, this may mean that the scientific objectives are not truly being met as this variability may contribute to difficulties in result interpretation and replication. Collection of replicates (*e.g*., triplicate samples for biological analysis) was included to help assess the amount of variability that existed within the selected sampling location. Within the BASALT program, scientific success was defined as the collection of replicate sets of samples over a set of EVAs that met *a priori* criteria for categorization as representative of specific alteration features relevant to defined scientific hypotheses listed in the STM. Examples of replicate sets collected at a station are described in the work of Stevens *et al.* (2019). Post-mission analysis is not considered at this time.

#### 2.4.2. Observational data collected during EVAs used to inform sample selection

During a deployment, the number of EVAs will be limited and at the same time, the amount of material that may be collected will also be limited. Prioritizing the scientific objectives within and across deployments is critical to ensuring that representative samples are collected that will enable testing of the hypotheses outlined in the STM. During the candidate sampling location search and pre-sampling phases of the BASALT EVAs, information was collected at regional, station, and location scales (as defined in Table 3) and used by the MSC-SST to assess the scientific value of one proposed sampling location as compared with another as informed by the STM. Selection of the observational data was an important component of the BASALT strategic planning phase, and the science team identified the type of observational information that would be most valuable for identifying high-priority sampling locations, drawing from long-term experience with field research. It was also used to identify Targets of Opportunity (TOPs) that could become a strategic priority in a later EVA. For a discussion of the decision-making process associated with these data products that occurred during an EVA, see the work of Stevens *et al.* (2019).

Terrestrial field scientists often triage samples to ensure that the samples used in subsequent analysis are optimal and representative of a site. They may have the option of discarding a sample while still at the field site if another “better” (more representative) sample is subsequently identified. In addition, Earth-based field teams may “oversample” and transport relatively large numbers (or sizes) of samples back to a lab for analysis and subsequent triaging after the main field collection has been completed. Such oversampling and triaging may not be possible on planetary missions that are subjected to sample weight and size constraints (Amador *et al.*, 2015). Field scientists working on the Earth will generally spend extensive amounts of time collecting contextual information about a scientific site (*e.g*., photos, measurements, detailed descriptive notes) used to aid in analytical result interpretation. These in-field observations gathered at the sampling site provide important site-specific details that may not always be garnered from other information sources of coarser resolution.

Collection of high-quality scientific samples is important if opportunities for multiple samples, and/or return to sample sites may be limited (or purposely avoided, *e.g*., to minimize cross-contamination). The interpretation of a field site and the samples collected from it rely heavily on the rationale used to select the samples in the field, and contextual observations provide important data that are critical to the interpretation of analytical results or identification of potential outliers.

#### 2.4.3. Commentary by EV crewmembers

The value of “scientists in the pilot seat” has been recognized in other analogs such as PLRP, where the observations and evaluations made by the scientist-pilot of the submersibles were critical to building up the contextual knowledge of the field site and in sample selection (Lim *et al.*, 2011). Constant commentary was also employed during the Desert Research and Technology Studies (D-RATS) field tests as a means of communicating valuable information regarding the field sites to the Science Backrooms (Hurtado *et al.*, 2013), and even the Apollo missions maximized time on station to enable opportunity for geological observations (as in Eppler *et al.*, 2013). Within BASALT, verbal observations and contextual descriptions provided by the EV crewmembers were deemed critically important pieces of information for use by the MSC-SST. Observations and descriptions are not only used to identify samples; these are also data products intended for use by the scientists during post-deployment analyses. Observational field practices are typically learned through experience rather than from formally established guidelines. Guidelines were created by the science team for EV and IV crewmembers as to critical observational data that should be gathered during different phases of the EVA based on experience with traditional field work. Verbal descriptive observations provided over the course of the EVA included:

- Weather conditions (wind speed, cloud cover, temperature, precipitation)
- General outcrop scale observations (*e.g*., orientation, shape, vegetation, etc.)
- Detailed outcrop and sample scale observations (*e.g*., color, vesiculation, primary minerals, friability) (see Stevens *et al.*, 2019)
- Environmental discomfort (*e.g*., exceptionally hot and uncomfortable)
- Potential TOPs (*i.e*., unexpected observations that may impact science objectives)
- Any other unexpected obstacles or conditions.

#### 2.4.4. Streaming video and still image protocol

Transmission of video and images has been identified by previous analogs as critical to informing science, not only during the deployments but also in post-deployment analysis where this type of contextual information is important for result interpretation (Lim *et al.*, 2011). Visual characteristics of alteration were among the primary tools that the EV crewmembers used to propose candidate sampling locations, for example, unaltered basalts were gray/black in color whereas highly altered, oxidized basalts were red (as described in the Observational Training Document). Therefore, visual information not only provided situational awareness but also facilitated collection of observational information and possible recognition of targets of scientific interest.

As part of BASALT strategic planning, the science team established protocols to guide the collection of video and still images at various points within EVA not only to inform scientific decisions during the EVA (see Payler *et al.*, 2019; Stevens *et al.*, 2019) but also to enable high-quality scientific results post-mission. In the BASALT EVA protocols, a video from each EV crewmember was streamed constantly when under a high bandwidth operational condition (Beaton *et al.*, 2017), providing both the IV crew and the MSC (under latency) with situational awareness of the EVA progression, important for tactical planning. Likewise, photographic images are data products whose usefulness is not limited to the actual deployments; they are among the most important contextual information collected in the field, and archived images are anticipated to be highly referenced during interpretation of analytical results.

**FIG. 1.**
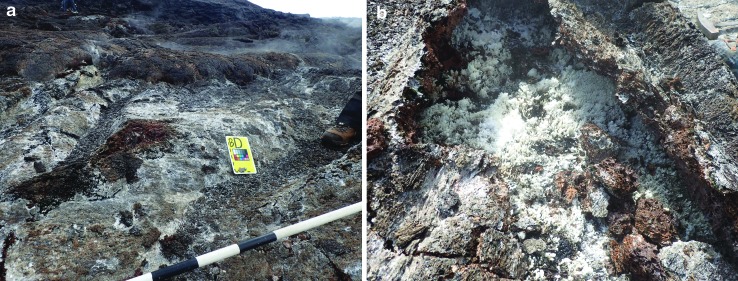
MD8 still images at candidate location BD showing an active fumarole, including **(a)** contextual image of fumarole with scale bar and **(b)** close-up image of fumarole opening showing white mineralization not visible at contextual image scale. MD, Mission Day.

A protocol was developed in the pre-deployment planning to determine the quantity and type of still images transmitted by the EV crew. This was strongly dependent on the bandwidth conditions of a given EVA, as under low-bandwidth conditions the number and resolution of images were limited, whereas under high-bandwidth conditions they were essentially unlimited (Beaton *et al.*, 2019a), but still presented a processing challenge to the MSC (example in Fig. 1). Drawing from previous field experiences, the team identified critical image types: (1) contextual images providing broad site and outcrop scale observation information as well as (2) close-up, high-resolution images of potential sample sites showing detailed features such as vesicularity and mineralization that would likely not be visible in the contextual images but provide important interpretative information about the samples (example in Fig. 1).

## 3. Results

The activities conducted during the BASALT EVAs were similar to what would occur during any field work campaign conducted on the Earth (*e.g*., identification of potential sample locations, collection of additional observational data, instrument scan, and, ultimately, sampling). However, BASALT EVAs also included spatiotemporal constraints designed to simulate Mars mission architecture (see Beaton *et al.*, 2017; Beaton *et al.*, 2019a). These constraints necessitated extensive pre-planning by the participating BASALT scientists before and during deployments to ensure that the scientific objectives were achieved. The STM described in the previous section was the scientific foundation referenced by the BASALT science team to plan EVAs executed during the two 2016 deployments.

### 3.1. Station identification and EVA traverse planning by the BASALT Science Team

Areas of interest within the two geographical zones of study (COTM and HVNP) were identified based on: (1) accessibility and safety, (2) scientific interest, and (3) potential for communications coverage (Lim *et al.*, 2019). Within each zone, km-scale regions of interest were identified that contain chemically diverse volcanic materials and alteration features (Highway Flow and Big Craters at COTM; Mauna Ulu at HVNP). In the months before deployments, the BASALT scientists used precursor satellite data to select EVA target stations (10-m diameter) and plan traverses within these regions (Table 3). Stations that were selected represent alteration types and features targeted in the objectives and hypotheses outlined in the STM relevant to recognizing potential microbial habitats. Parameters of interest that may have relevance for habitability included mineralogy, texture, and secondary mineral composition due to aqueous alteration (Benson *et al.*, 2011; Hausrath and Tschauner, 2013; Kelly *et al.*, 2014). Details of the association of these parameters with the alteration types identified as of interest in the STM are outlined in the work of Hughes *et al.* (2019).

Visible wavelength (aerial) imagery at a resolution of ca. 0.15 m per pixel and multispectral imagery at a resolution of ca. 2 m per pixel (Beaton *et al.*, 2017; Beaton *et al.*, 2019a) were used to refine station selection and identify visible alteration features that would meet the STM objectives. Multispectral imagery (Table 2) was used to select a variety of stations that represented a gradient of alteration ranging from high to low. The multispectral imagery, rather than the aerial imagery, often played a more prominent role in identifying alteration features of scientific interest to the BASALT scientists. The multispectral imagery provided data regarding geochemical variability that was not captured in the aerial imagery but from a scientific perspective was critical in selecting targets that were expected to show distinct alteration features; aerial imagery alone did not suffice. Examples of aerial and multispectral imagery used as part of station selection are shown in Sections 3.1.1 and 3.1.2.

The selected 10-m diameter stations dictated EVA traverse planning and strategic prioritization of EVA execution within the deployments. The 10-m diameter range was chosen to provide a search area that was large enough to find desirable features (*e.g*., alteration types) based on the available precursor imagery. Each individual 10-m station was nominally anticipated to contain outcrops of the distinct end-member alteration features identified as of interest in the STM (*e.g*., unaltered versus highly altered basalts). In doing so, the EV crewmembers were given some level of flexibility to search for candidate sampling locations within loosely defined boundaries to allow multiple stations to be visited in a single EVA. Stations were grouped to reduce traverse time between stations while promoting coverage of a specific area.

#### 3.1.1. Craters of the Moon National Preserve and Monument

**FIG. 2.**
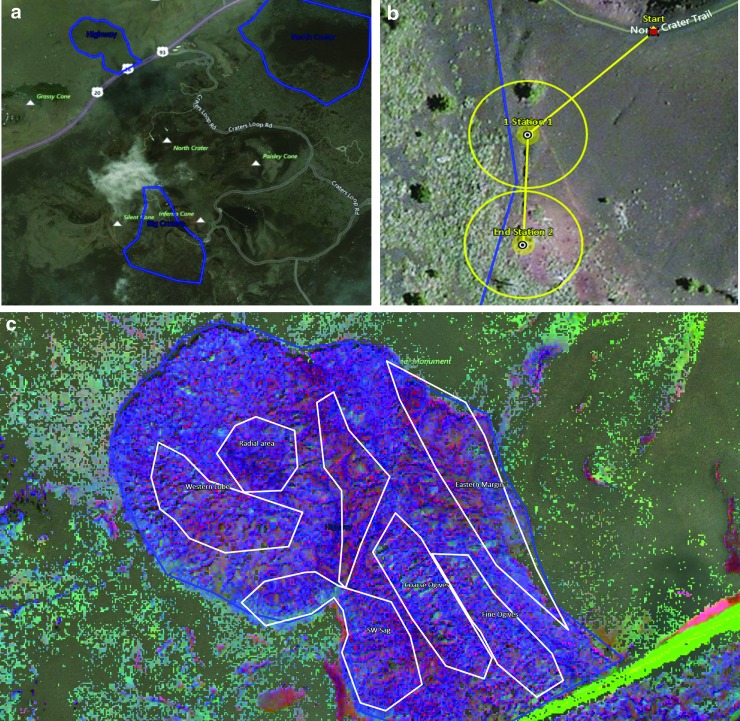
Visible wavelength (aerial) imagery within xGDS of **(a)** COTM showing regions of interest Highway Flow and Big Craters (outlined in blue). **(b)** Aerial imagery of the gas crack feature identified within the Big Craters region and selected stations and EVA traverse plan. **(c)** Multispectral imagery of Highway Flow with “theme” areas and illustrating dark, blue/purple colors expected to indicate a lack of oxidation and thus minimally altered basalts. The lack of oxidized material is especially dominant in the western section of the region and targeted stations were identified in this section that were predicted to nominally contain unaltered basalt features that could be identified by the EV crew. COTM, Craters of the Moon National Monument and Preserve; EV, extravehicular; EVA, extravehicular activity; xGDS, eXploration Ground Data System.

Within the COTM region, as part of the BASALT-1 pre-deployment strategic planning, the science team focused on selecting stations representative of alteration end-members: basalts exhibiting high degrees of alteration and unaltered basalts spread across two distinct flows (Highway Flow and Big Craters) (Fig. 2) with different geochemical make-up. Further details on the geochemical composition of the two flows of interest at COTM are provided in the work of Hughes *et al.* (2019). Alteration features were identified by using a combination of available aerial imagery and multispectral data archived and accessible via *Minerva*. Within these two flows, themed areas (*e.g*., “Alteration Corridor” where it was anticipated that a gradient of alteration would be easily identifiable by the EV crew across multiple stations) were created by the science team that were used to represent general morphological, geological, and/or geochemical features that were of scientific interest.

During station selection, science team members selected stations within these themes that could be grouped together to provide multiple targets to select from and meet EVA priorities. For example, within the Big Craters region, a gas crack was observed in the aerial imagery that was expected to be associated with basalts exhibiting high degrees of alteration (Fig. 2b). The science team, therefore, selected stations near this geological feature that would form the targets for one EVA during the deployment. Degrees of basalt alteration were also hypothesized based on the multispectral imagery, for example, bright coloration was anticipated to represent high degrees of alteration whereas contrasting dark, blue/purple colors were anticipated to reflect unaltered basalt (Fig. 2). Station selection was a collaborative effort among SST members via teleconference and synchronous use of xGDS based around STM objectives and hypotheses that ultimately resulted in ca. 25 stations distributed across the two COTM flows that were investigated over the course of 10 In-field, In-Sim mission days (MDs) (see Lim *et al.*, 2019, for details). During the first deployment at COTM, two stations were always grouped together with the intention of collecting three full sample suites at each station.

#### 3.1.2. Hawai‘i Volcanoes National Park

**FIG. 3.**
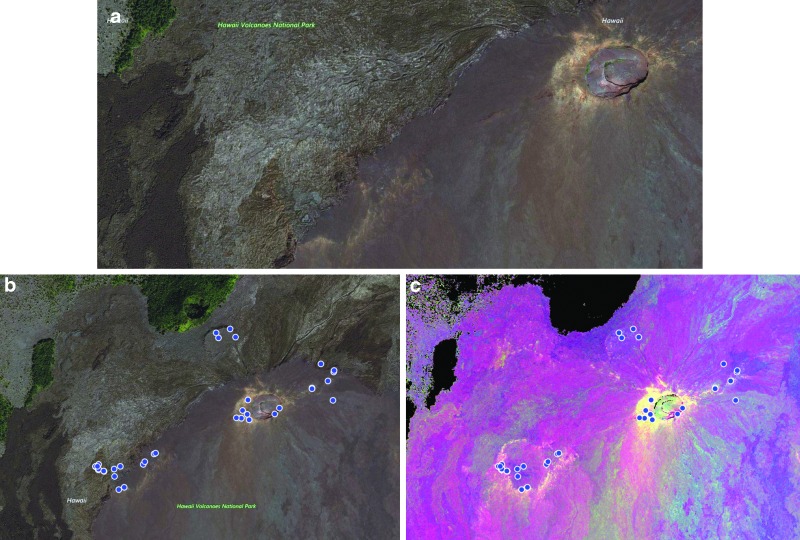
Aerial imagery within xGDS and used by the science team for EVA traverse planning of **(a)** Mauna Ulu, HVNP. Proposed HI_2016 stations (blue dots) within Mauna Ulu region based on precursor satellite aerial imagery **(b)** and multispectral imagery **(c)**. Stations were selected based on identification of features of scientific interest (*e.g*., fumaroles) incorporating input from all science team members to create an initial set of proposed stations that met the minimum criteria for alteration features and number of sample suites required for minimum success. HVNP, Hawai'i Volcanoes National Park.

**FIG. 4.**
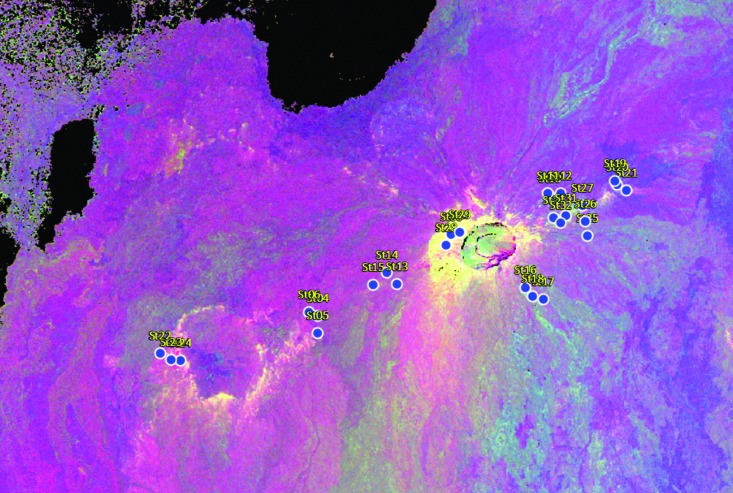
Actual HI_2016 stations used during deployment. Initial targets of interest were refined based on scientific priority and logistical constraints (clustered stations were selected to minimize translation times, see Beaton *et al.*, 2017). Bright features (white/yellow) were interpreted to represent oxidized material whereas darker blue/purple represent lower degrees of alteration.

The targeted search areas for each EVA was expanded to three stations for BASALT-2 in HVNP based on the scientist's desire to expand the search area. As part of the strategic planning that occurred in the months before the 2016 HVNP deployment, each member of the science team used the available aerial and multispectral imagery to identify potential stations within the Mauna Ulu region that best met the proposed science objectives (Fig. 3). For example, stations that showed bright white coloration in the multispectral map were interpreted as reflecting evidence of oxidized material, thus representing a highly altered end-member. Features of interest were expanded from COTM to include active fumaroles, associated with bright coloration in the multispectral imagery, again suggestive of the presence of oxidized material. From an initial selection of ca. >65 stations, the stations ultimately used for EVA traverse planning were prioritized by the team in the months leading up to the deployment to ca. 30 that could be visited within the 10-day deployments (1 EVA per day) (Figs. 3 and 4). Stations were refined by science team consensus through removal of redundant locations (*i.e*., identical station locations selected by multiple team members), iterative discussions regarding confidence in the interpretation of alteration features leading to the elimination of some of lower confidence, and the need for a minimum number of stations reflecting alteration end-members identified by the science team to enable post-deployment analysis and hypothesis testing. As alteration features of interest were typically identified in multiple locations across the larger region (km scale), it was determined by the science team that an assessment of the spatial heterogeneity that existed across the single overall region (*i.e*., Mauna Ulu) was an important and relevant science question. Therefore, as part of the station selection process, the science team decided to select stations with representatives of each alteration feature that were widely distributed across the geographic region to provide some indication of geographical variability (Fig. 4).

### 3.2. EVA objective prioritization

**FIG. 5.**
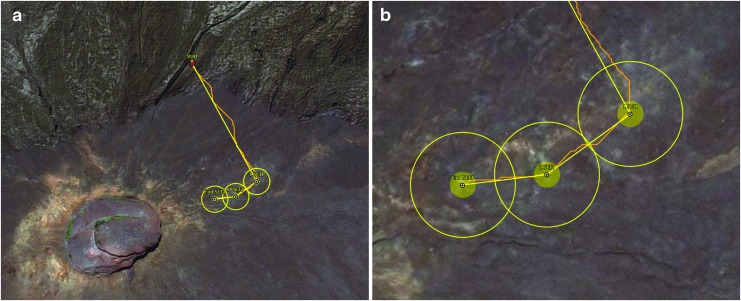
Example of EVA traverse plan for MD9 at Mauna Ulu showing **(a)** three selected stations of interest and EVA start location and **(b)** a close-up image showing the 10-m diameter station (small, filled yellow circle) and larger boundary zone used to indicate to the EVA crew that they were approaching the station boundary. The yellow line represents the proposed traverse path.

The scientists recognized during the planning process that some features (*e.g*., active vs. relict fumaroles in Hawai‘i) were challenging to identify based on the precursor data available, and likely required ground-level observations. Therefore, as part of the strategic planning, the science team prioritized EVA objectives based on the primary science objective pursued in each one. For example, at Mauna Ulu, pre-deployment station selection for hotter (>70°C), active fumaroles might be challenging because neither aerial imagery nor multispectral imagery provided any temperature information. Instead, this was a target that the EV crewmembers would need to identify on the ground by using visual observations and hand-held instruments such as the FLIR that provide temperature readings. As hot, active fumaroles were considered by the scientists to represent a high-priority science target, EVAs with this target objective were scheduled early in the deployment to allow future strategic and tactical re-planning of EVAs later in the deployment if this objective was not achieved (see Section 4.2) (Stevens *et al.*, 2019). Within a given EVA, the visitation order of the stations was also prioritized so that the first two stations represented the highest priority EVA objective and the third station represented the secondary objective of the EVA (Fig. 5). It was anticipated that the third station assessment and feedback provided by the MSC-SST would be more limited relative to the other two stations due to the latency and associated EVA architecture (Beaton *et al.*, 2019a).

To allow for some flexibility in responding and adapting to new discoveries during an EVA (Hodges and Schmidtt, 2011), TOPs as introduced at PLRP (Lim *et al.*, 2011) were included in the flight rules if EVA crewmembers (or potentially the MSC-SST) observed a feature of interest during the traverse, station approach, or potentially within the station that was not identified as a specific EVA objective. Although not necessarily a primary objective of the active EVA, a TOP may address a major scientific goal of the entire program as outlined in the STM. As such, it may become a top priority objective, potentially superseding the original objective as decided by the EV crewmembers and/or SST. Field scientists on the Earth are often able to modify traverse plans based on ground-level observational data (*e.g*., obstacles, opportunistic samples) to best meet scientific objectives. Such flexibility may entail not only modifying individual plans to meet objectives but also changing overall EVA or deployment objectives in response to new information if in-field observations or findings open up new scientific questions and hypotheses (Eppler *et al.*, 2013; Hurtado *et al.*, 2013). In the event of a TOP identification, the EVA crewmembers could deviate from the prescribed EVA plan for follow-up by using a predetermined set of rules (*e.g*., time allowed for photos). Information gathered could then be used to determine whether action would be taken during that EVA or on a future EVA.

### 3.3. Daily EVA briefs

**FIG. 6.**
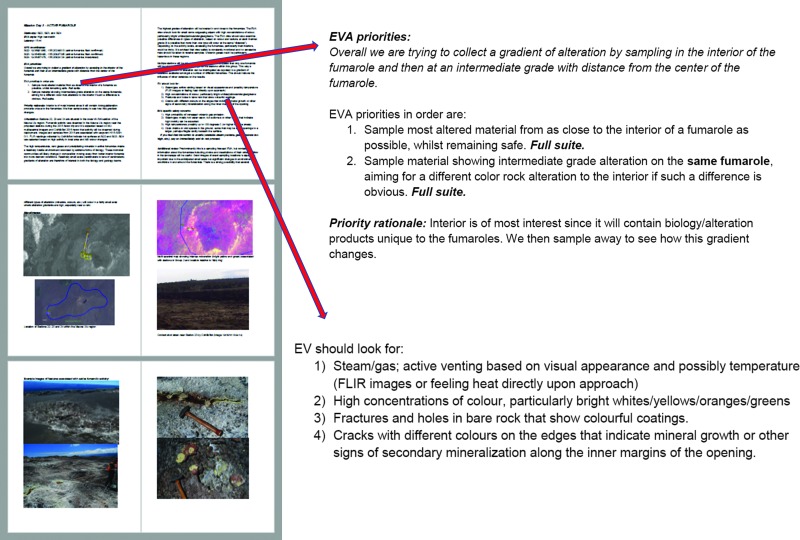
Daily mission brief for MD3 in Hawai‘i illustrating EVA priorities, including the rationale for this priority and features that the EV crew should look for that will aid in identifying the alteration feature of interest. EVA traverse plans, station maps (aerial and multispectral) are also included as are example photographs showing specific alteration features, in this case for a relict fumarole.

The strategic planning process resulted in daily briefings that outlined the scientific priorities associated with the individual EVAs. The specific objectives and priorities of each EVA were stated based on the STM, in addition to supplemental details that provided guidance on the identification and collection of samples representative of the key features of interest to the scientific team (Fig. 6). The EVA briefings were distributed via e-mail to the entire crew the day before the EVA; it was decided not to distribute them further in advance to allow for flexibility in changing EVA priorities as the deployments evolved and sample types were collected (or, were not collected, see Section 4.2). The most recent version of each document was also included in *Minerva* (Marquez *et al.*, 2019) for quick access by any member of the team. Briefings for each EVA were finalized the day or two before the assigned execution day since scientific objectives were reordered in response to progress made during EVAs performed earlier in the deployment. As an example, Figure 6 illustrates an excerpt of an EVA priority description from HI_2016 MD3, to identify and collect a sample suite from an active fumarole and a secondary sample of a location a short distance away from the main vent. As part of the mission briefs, the EVA priority and rationale was combined with a detailed description of the stations that were to be explored (including precursor images, or orbital imagery, if appropriate, to provide context) as well as guidelines as to what the EV crewmembers should be looking for during the candidate location search (Fig. 6). The daily mission briefs specific to each EVA were critical to ensuring that the EV crewmembers, acting in concert with the MSC-SST, could maintain a clear understanding of the objectives and make informed suggestions for candidate sampling locations.

## 4. Discussion

### 4.1. Modification of EVA objectives

At COTM, the ordering of the EVAs within each region was based on the scientific objectives and targets expected at the two distinct flows of interest. Although only a single region was being targeted at HVNP, it was recognized that though the EVA order was initially based on scientific priority, the logistical constraints of moving the communications equipment meant that the EVAs could not be done in the identified pre-deployment order originally selected by the science team. Instead, at the onset of the Hawai‘i deployment, it was necessary to reconsider the order of the schedule of EVA objectives; moving from a priority order that was based on scientific priority alone to one that better facilitated the necessary movement of operational equipment required while still maintaining the scientific objectives. Re-assessment of the order of EVAs was based on a re-prioritization of given target stations within a specific communications region at Mauna Ulu. For example, accomplish all EVAs on the eastern flank of Mauna Ulu before moving communications infrastructure. Although this created an inherent risk of not obtaining samples of high scientific priority early in the mission (depending on the location of stations of highest scientific priority), the BASALT science team selected and grouped the stations such that the minimum success threshold of two full sample suites per alteration type could still be achieved within a more limited geographic location if necessary (*e.g*., representative samples of each alteration type were collected in one communications coverage area before equipment relocation).

### 4.2. Tracking scientific progress during the deployments

For each deployment, the scientists defined the number of sample suites of each type of alteration feature that were necessary to conduct the laboratory-based analyses to produce results that satisfy the requirements outlined in the STM and are suitable for peer-reviewed publications. The minimum threshold for scientific success of a deployment was defined by the SST as the collection of at least two sample suites from alteration end-members within the four categories previously outlined. To ensure that science objectives were being met both within and between deployments, the scientists tracked daily scientific success and developed strategic responses to each EVA outcome. To acquire the samples and data needed to address scientific questions and hypotheses, it was important to assess whether daily EVA samples met the criteria for post-mission scientific analysis (*e.g*., was the sample collected representative of the feature of interest) and whether the correct number of replicates were collected over the course of the individual deployments.

Samples collected during EVA were regularly assessed post-EVA (*e.g*., visually inspected, re-scanned by using hand-held instruments) by members of the science team, and a consensus decision was reached as to whether that sample met the targeted alteration style, that is, whether the EVA objective had been met. In some cases, it was determined by the collective expertise of the scientific team that a collected sample did not meet the criteria for a specific alteration type and that a future EVA must again target that alteration feature. For example, samples identified as “unaltered” and collected in early MDs at COTM were deemed to have some degree of alteration (*e.g*., secondary minerals) on closer inspection post-EVA. Although they may have met the criteria of most unaltered material present within a given station, they were not an ideal example of unaltered material and, thus, did not necessarily meet the overall science objective of obtaining an unaltered end-member.

**FIG. 7.**
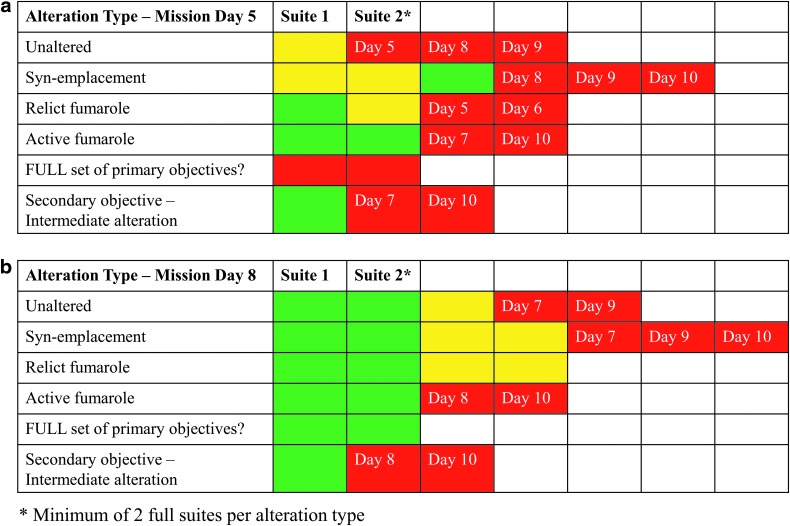
Example of the daily strategic matrix, as presented in the daily mission brief for MD5 **(a)** and then updated on MD8 **(b)** that was used to track progression toward obtaining a minimum of two replicate sets of representative samples of different alteration types. Green shading indicates that representative end-members have been obtained. Yellow indicates that an example of this alteration type has been collected that while meeting the minimum criteria for this alteration type, is not an optimal end-member example. Red indicates that appropriate samples have not been collected. The projected EVA days that will have the associated alteration types as primary objectives are listed. Within this example framework, the minimum scientific success of two replicates of the primary alteration types was assessed as having been achieved by MD8. However, a secondary objective (only to be accomplished if primary objectives are met) remains to be fulfilled but is planned for completion in the MD8 EVA.

A daily sample-type matrix (DSM) was used to track the sample type, number of replicates collected, and was updated daily after each EVA was completed and samples were assessed by the science team. Samples were color coded to indicate whether replicate sets were outstanding, acceptable but not optimal, and optimal (Fig. 7). An updated version of the DSM was presented at the daily evening team debriefs to ensure that the entire science team was aware of the progress being made toward the overall scientific objectives during the deployment. It was also included in the daily mission brief and contained notation indicating EVA MDs assigned to outstanding alteration targets (Fig. 7). The evolution of this daily sample matrix and ties to the overall STM determined whether EVAs planned before the mission were re-planned and/or re-ordered to ensure that all necessary features and replicates were collected. The DSM as shown in Figure 7 from the daily mission brief for MD5 indicated that the two targets for that EVA were Unaltered and Relict Fumarole. By MD8, the matrix was updated to show that the minimum number of replicates of each alteration type had been obtained and the EVA objectives were refined to collect additional suites of certain sample types of interest (*e.g*., active fumarole). If all primary objectives were met (a minimum of two full sample suites for each alteration type), EVAs were planned to obtain additional sets and/or address secondary deployment objectives.

As the scientific progress of the daily activities were evaluated over the course of the deployment, EVA objectives gradually became more specific as minimum thresholds were met, although early collected samples tended to be skewed toward features that were easier to locate. It eventually became necessary to target sample locations and types that met specific parameters with little room for deviation and limited secondary priorities to ensure adequate scientific progress as pre-determined by the STM. As an example, a comparison between two end-member alteration types is not possible if samples representative of one of the end-members cannot be found within the geographically selected areas where the EVA is executed. As such, the daily EVA briefs became progressively more focused on obtaining specific alteration types that were outstanding based on the DSM, and more often aimed at addressing secondary science objectives as primary ones were met.

Primary priorities of each EVA as determined before the deployment could not always be met within selected station(s), impacting scientific productivity and leading to the need to re-plan EVA objectives to meet outstanding objectives. In those circumstances, the BASALT SST had to decide whether to prioritize the scientific objectives already ascribed to a future EVA, or to re-plan or re-order the future EVAs to meet the missed objectives. These decisions were facilitated by examining the overall deployment goals as outlined in the STM and ensuring that the (re)planning of the remaining EVAs would enable collection of the sample suites needed to adequately address the scientific hypotheses. The figure mentioned earlier showing the evolution of the DSM demonstrates how specific MD EVAs were assigned priority targets as of MD5, but that these targets were adjusted, and EVA MDs were re-assigned by MD8 due to strategic re-planning by the science team. The MD briefs also evolved over the course of the deployments due to this need for re-planning. Within the briefs, both primary and secondary objectives were listed that would allow the teams to continue to address the overall mission objectives as outlined in the STM if during any given EVA, the primary objective could not be met. In some cases, precursor imagery of insufficient resolution hindered our ability to identify the targets of interest (*e.g*., distinguishing active vs. relict fumaroles). In these situations, having secondary targets within the EVA objectives allowed the team to collect samples that would either still target one of the other alteration types of interest and/or be of scientific use. Secondary objectives, such as the collection of samples of intermediate fumarolic alteration, were pre-determined based on the STM to ensure that their collection would still enable scientific analyses and hypothesis-driven research.

As mentioned earlier, the primary objective of any given EVA was not always met. For example, identification and collection of a relict fumarole was a primary objective on MD5 in Hawai'i. However, no relict fumaroles were present within the targeted station being explored during that EVA. As a result, the MSC-SST made the strategic decision during the EVA to instead target another overall mission objective of collecting a syn-emplacement sample that was present in the station. The EVA conducted on MD6 was modified to include Relict Fumarole as a primary target to ensure that the minimum number of sample suites required for scientific success was achieved. Targeted collection of a hot fumarole (ca. 70°C) was also a primary objective of planned EVAs in Hawai`i. However, although the precursor imagery allowed for some estimation of the presence of fumaroles, this type of imagery did not allow for a pre-deployment estimate of ground temperatures that could only be obtained once crewmembers were on site. Initial EVAs with “hot fumarole” as a primary objective were not able to identify targets that met these specific temperature constraints. Instead, early Hawai`i mission EVA sampled fumaroles that were closer to ca. 60°C, considered to be acceptable by the science team if no hotter ones were found (hence, meeting the minimum success threshold in the daily sample matrix figure depicted earlier). This decision was made to ensure that examples of this alteration type (active fumarole) were obtained, regardless of temperature, to enable the scientists to address the hypotheses in the STM. It was recognized that by doing so, post-deployment analytical results and any trends observed must take into consideration that the collected samples were of less extreme temperatures than initially targeted. Only one EVA successfully identified and sampled a fumarole that was ca. 85°C (MD3) during the deployment. As the scientific objective identified pre-mission was to obtain samples representing at a minimum two, but ideally three distinct fumaroles, this specific objective was still outstanding at the end of the planned EVA simulations. Instead, an active fumarole of 72°C was identified in an area of HVNP that was not targeted during the simulation and sampled by a small out-of-simulation field team. As achieving this specific temperature objective required exploration and sampling outside of the simulations, this highlighted the need for better and more relevant precursor information for EVA station selection (*e.g*., thermal imaging maps) and may also have benefited from increased flexibility and spatial freedom within the structure of an EVA to venture out of the pre-selected stations to best meet the scientific objectives.

### 4.3. Informing future planetary science strategic planning

The following insights were derived from our experiences during the 2016 BASALT deployments that aim at informing future deployments to best enable scientific return and provide key information that may be used to construct future Mars EVAs that incorporate astrobiological sampling.

#### 4.3.1. High-resolution precursor data tailored to the scientific objectives are critical to mission success

Precursor information available to strategic planning teams must be targeted and relevant to the scientific questions and hypotheses to be investigated. As such, the selection of precursor imagery must be informed by the STM before deployments. There is an increased probability that scientific objectives will be met in a predetermined region/station if the precursor information made available before the mission is of the highest resolution and relevance available. The 0.15-m per pixel aerial imagery used to plan the EVAs during the BASALT 2016 deployments was not always of sufficient resolution to adequately identify features, and although the multispectral data available for the regions gave indications of sites of high alteration, they were of lower resolution and, therefore, less useful in directing EV crewmembers to specific locations.

Although nominally the expected features of scientific interest were present within the stations pre-selected by the science team, in some cases they were either not present at all (*e.g*., anticipated active fumarole was actually relict) or not appropriate for meeting the EVAs scientific objectives (*e.g*., fumarole may be present but not of high temperature). A minimum resolution of 0.15-m per pixel was needed; however, higher resolution would have been more optimal for identifying and targeting features of interest. It quickly became apparent to the BASALT science team that base maps of the highest resolution possible are critical to ensuring that EVAs traverse plans are efficient and effective in targeting stations that will best meet the scientific objectives. Further analysis of the resolution requirements is in the work of Beaton *et al.* (2019b). The existence of a detailed thermal map of the HVNP field region would have proved invaluable for selecting stations more likely to contain fumaroles within the temperature range of interest.

One method used to mitigate this issue was the use of secondary (back-up) objectives as employed in the BASALT deployments. However, this can lead to the need for strategic re-planning during the mission if high priorities are missed. If secondary objectives consistently become primary objectives, overall scientific progress can be hindered. For example, as the secondary objectives often consisted of volcanic features that were easier to find relative to the primary ones, this meant that collection of replicate samples from certain features was rapidly achieved in the early EVAs, whereas the more challenging targets remained unfulfilled. As detailed earlier, to ensure that all targeted materials identified in the STM were collected, the EVA objectives at the end of the mission became more targeted and focused on a specific alteration type rather than allowing for some flexibility in finding other types. As the deployment progressed, achievement of primary objectives of the EVA was necessary to ensure a successful deployment that met the scientific objectives determined pre-mission.

A related aspect is that from a temporal perspective, the amount of time for the science team to provide an assessment of whether the samples collected met the pre-mission criteria for alteration types (and, thus, were considered a successful collection) was limited post-EVA, especially at COTM. Although the time allocated was improved at HVNP, dedicating time for the science teams to evaluate scientific success of EVAs and strategically re-plan traverses if needed is recommended for future planetary EVA mission operations.

#### 4.3.2. Scientific discovery is an evolutionary process

Regardless of the effort involved in the strategic planning for an EVA or deployment, if selected stations do not meet the strategic objectives then the EV crew must be able to adapt—either by expanding their search area or by having alternative objectives that can be identified and accomplished instead. As part of the BASALT strategic planning, primary and secondary objectives were identified within each EVA to ensure that data and samples were being collected that met the overall objectives of the project even in situations where the anticipated primary targets were not present within selected stations. As recognized in other analogs, adaptability is critical in enabling the science team to respond to unexpected or scientifically interesting findings that may ultimately address a high-level question identified in the STM (*e.g*., D-RATS) (Hodges and Schmitt, 2011). When it was recognized that the pre-selected 10-m stations did not always contain the features of scientific interest, EV crewmembers were given some leeway with respect to venturing outside of the station boundary during the candidate location search to find primary targets. Although not employed often, the ability to assign TOPs gave the EVA crew some level of freedom in identifying features of potentially high scientific value based on the STM that were not restricted to the predetermined paths and stations. The TOPs will continue to be part of the BASALT EVA protocols.

## 5. Conclusion

EVA operations pursuant to non-simulated scientific objectives during the 2016 BASALT deployments provided opportunities to develop and study the strategic planning process required to integrate human scientific data collection and sampling activities. Coordinating the planning effort was the generation and adaptation of scientific hypotheses into an STM that was used to track and articulate the associated EVA objectives necessary to satisfy scientific questions. Results suggest that creation of a foundational STM is a necessary initial step of the strategic planning process of science-driven future planetary missions. The STM facilitated the translation of scientific questions into actionable objectives and tasks used to drive the EVA operations. Formulation of an STM by a multidisciplinary science team through an iterative process before EVA planning was critical to accomplishing scientific objectives and responding to new data and findings during a deployment. The STM also served as a reference point during the simulations to track scientific success (acquisition of samples) and to strategically replan EVA objectives when necessary to satisfy primary objectives.

The BASALT simulations highlighted that relevant and high-quality (*i.e*., high resolution) precursor imagery tailored to specific scientific objectives is necessary to effectively strategically plan EVAs. Thermal maps of the Mauna Ulu region would have aided in identifying active versus relict fumaroles. The inclusion of secondary objectives within an EVA plan was important for ensuring overall deployment and mission success with respect to achieving scientific objectives if primary objectives were not readily attainable. The inability to achieve primary scientific objectives during an EVA due to insufficient precursor information during the planning stages was problematic and brings to light challenges that may be faced in planning future planetary missions. Future planetary missions need to prioritize capturing and creating data products that enable selection of targets and planning of EVA traverse paths that have a high probability of meeting the set scientific objectives of not only the individual EVAs but also the entire deployment. Lessons learned from the 2016 BASALT deployments are currently being applied to the planning of the 2017 deployment during the preparation of this article.

## Abbreviations Used

BASALT: Biologic Analog Science Associated with Lava Terrains
COTM: Craters of the Moon National Monument and Preserve
D-RATS: Desert Research and Technology Studies
DSM: daily sample-type matrix
EV: extravehicular
EVA: extravehicular activity
FLIR: forward-looking infrared
HVNP: Hawai‘i Volcanoes National Park
IV: intravehicular
MD: Mission Day
MER: Mars Exploration Rovers
MSC: Mission Support Center
PLRP: Pavilion Lake Research Project
PSTAR: Planetary Science and Technology Through Analog Research
SAP: Science Activity Planner
SST: Science Support Team
STM: Science Traceability Matrix
TOPs: Targets of Opportunity
xGDS: eXploration Ground Data System
